# Tannic acid as a plant-derived polyphenol exerts vasoprotection via enhancing KLF2 expression in endothelial cells

**DOI:** 10.1038/s41598-017-06803-x

**Published:** 2017-07-27

**Authors:** Yanni Xu, Peng Liu, Suowen Xu, Marina Koroleva, Shuya Zhang, Shuyi Si, Zheng Gen Jin

**Affiliations:** 1 Aab Cardiovascular Research Institute, Department of Medicine, University of Rochester School of Medicine and Dentistry, Rochester, NY 14620 USA; 2 Institute of Medicinal Biotechnology Peking Union Medical College and Chinese Academy of Medical Sciences, Beijing, China; 3 Key Laboratory of Fertility Preservation and Maintenance of Ministry of Education, Department of Biochemistry and Molecular Biology, Ningxia Medical University, Yinchuan, China

## Abstract

The transcription factor Kruppel-like factor 2 (KLF2) is a critical anti-inflammatory and anti-atherogenic molecule in vascular endothelium. Enhancing KLF2 expression and activity improves endothelial function and prevents atherosclerosis. However, the pharmacological and molecular regulators for KLF2 are scarce. Using high-throughput luciferase reporter assay to screen for KLF2 activators, we have identified tannic acid (TA), a polyphenolic compound, as a potent KLF2 activator that attenuates endothelial inflammation. Mechanistic studies suggested that TA induced KLF2 expression in part through the ERK5/MEF2 pathway. Functionally, TA markedly decreased monocyte adhesion to ECs by reducing expression of adhesion molecule VCAM1. Using lung ECs isolated from *Klf2* ^+/+^ and *Klf2* ^+/−^ mice, we showed that the anti-inflammatory effect of TA is dependent on KLF2. Collectively, our results demonstrate that TA is a potent KLF2 activator and TA attenuated endothelial inflammation through upregulation of KLF2. Our findings provide a novel mechanism for the well-established beneficial cardiovascular effects of TA and suggest that KLF2 could be a novel therapeutic target for atherosclerotic vascular disease.

## Introduction

Kruppel-like factors (KLFs) are members of the zinc-finger transcription factors family that are critical in the regulation of cell proliferation, differentiation and inflammation^1, 2^. So far, the mammalian KLF family has 17 members^3^, each has 3 contiguous C2H2 (cysteine-histidine) type zinc fingers at the carboxyl terminus^4, 5^. In particular, KLF2 can modulate expression of genes critical in regulating endothelial homeostasis, vascular tone, thrombosis and inflammation, and has been regarded as an atheroprotective factor^2, 6, 7^. KLF2 is potently induced by laminar shear stress and confers anti-inflammatory and anti-thrombotic effects on the endothelium^7, 8^. KLF2 strongly induces endothelial nitric oxide synthase (eNOS) expression, but decreased vascular cell adhesion molecule 1 (VCAM1) and endothelin-1 (ET-1) expression^4, 9–11^. Endothelial KLF2 expression is regulated by the MEK5/ERK5 (dual specificity mitogen-activated protein kinase 5 (MEK5)/extracellular-signal related kinase 5 (ERK5) pathway requiring myocyte enhancer factor 2 (MEF2) transcription factor, which mediates beneficial effects of laminar flow^12–14^. Our previous studies showed that KLF2 was also negatively regulated by histone deacetylase 5 (HDAC5)^15, 16^. *In vivo* animal experiments demonstrated that endothelial cell-specific deficiency of KLF2 predisposed to atherosclerosis development^17^. Hemizygous deficiency in Klf2^+/−^mice on apolipoprotein E deficient (*ApoE* ^−/−^) background (*Klf2* ^+/−^; *ApoE* ^−/−^) exhibited increased diet-induced atherosclerosis versus wild-type ApoE deficient mice (*Klf2* ^+/+^; *ApoE* ^−/−^)^2^. In addition, myeloid-specific Klf2 knockout in an atheroprone LDL receptor-deficient background (*Ldl* ^−/−^) increased atherosclerosis progression^18^.

Based on these studies, modulating KLF2 expression or function could be a novel strategy for the prevention and treatment of inflammatory related disease including atherosclerosis^2, 6, 9^. Thus, the aim of this study is to screen small-molecule activators that modulate KLF2 expression. Based on luciferase-based assay, we identified that a small molecule drug tannic acid (TA) was a novel KLF2 activator. TA is a specific commercial form of tannin (a type of plant polyphenol). Previous studies showed that TA reduced aortic lesion formation in *Apo* ***E*** ^−/−^ mice^19^. However, the anti-inflammatory action of TA in ECs has not yet been investigated. In the present study, we provide evidence that TA induces endothelial KLF2 expression and thereby attenuates vascular endothelial inflammation. Collectively, our findings not only identified TA as a novel KLF2 activator but also demonstrated that KLF2 could serve as a promising therapeutic target for the treatment of atherosclerosis.

## Materials and Methods

### Cell culture

COS-7 cells (ATCC, Rockville, MD) were cultured in DMEM (Corning, Cellgro®, USA) containing 10% fetal bovine serum (FBS) (Gibco). HCAECs (#300K-05A, Cell Applications Inc., San Diego, CA) were cultured in Meso Endo Cell Growth Media (Cell Applications Inc.) containing 10% FBS and Growth Supplement (#212K-500, Cell Applications Inc.)^20^. Passage 4-6 of HCAEC cells were used for experiments. Human umbilical vein endothelial cells (HUVECs) were isolated from umbilical cords from normal pregnancy women in accordance with the University of Rochester human subjects review board procedures that prescribe to the Declaration of Helsinki^20^. Human umbilical cores are obtained under informed consent from all participants and conform to HIPAA standards to protect the privacy of the donor’s personal health information. HUVECs were cultured in Medium 200 (#M-200-500, Thermo Fischer Scientific, Waltham, MA) containing 10% FBS and low serum growth supplement (LSGS) (#S-003-10, Thermo Fischer Scientific). Passages 3–6 of HUVECs were used for experiments. THP-1 cells (ATCC) were grown in RPMI 1640 containing 10% FBS. Lung ECs were maintained in DMEM containing 20% FBS, penicillin/streptomycin and heparin. All cells were cultured at 37 °C with 5% CO_2_ in cell incubator.

### Cell transfection and luciferase reporter gene activity

The drug library was obtained from University of Rochester Pathway Discovery Resource (NCI Spectrum Compound Library containing 2400 chemical compounds, 1 mM for every compound). The -1.7-kb *KLF2*-luc promoter-driven luciferase reporter (KLF2-luc) plasmid, KLF2 -221bp promoter wild-type (WT) and KLF2 -221bp promoter mutant plasmids were gifted by Prof. Mukesh Jain^21^. KLF2 -221 plasmid contains MEF2 binding site while in KLF2 -221 mutant plasmid, the MEF2 binding site was mutated.

KLF2 luciferase assay was performed as follow. Briefly, COS-7 cells at a density of 80–90% in 100-mm dish were transfected with 5.4 µg plasmid (KLF2-luc or KLF2 -221 WT or KLF2 -221 mutant plasmid) using lipofectamine 2000 (Thermo Fisher Scientific, USA) in Opti-MEM (Gibco) for 6 h. Then the transfected cells were grown in 96-well plates (3.5 × 10^5^ cells/well, 100 µl/well). After 6 h, 1.0 µl /well drugs at a final concentration of 5 µM in 200 µl medium/well were added and incubated for 24 h. The KLF2 luciferase reporter gene activity was detected using Promega dual-luciferase reporter 1000 assay system in a microplate spectrophotometer (BMG, USA). The luciferase activity of the tested compound is as calculated as compound firefly luciferase value/DMSO firefly luciferase value.

### RNA isolation and quantitative PCR (qPCR)

Total RNA extracted from cultured HCAECs or lung endothelial cells (ECs) were performed as previously described^20^. TA was purchased from Sigma-Aldrich Co. (#MKBV0516V). Cells were grown in 24-well plate and treated with TA (0, 0.1, 1, 10 and 20 µM) for 24 h. Total RNA from cells was extracted using a QIAGEN RNeasy Mini kit (Qiagen) and converted into complementary DNA (cDNA) using a High-Capacity cDNA Reverse Transcription Kit (#4374966, Applied Biosystems, Foster City, CA). Quantitative real-time PCR (qPCR) was performed in a Bio-Rad iQ5 real-time PCR thermal cycler using iQ SYBR Green Supermix (#1708886, Bio-Rad). Relative mRNA expression of target genes was normalized to GAPDH^22^.

### Western blot analysis

Western blot was performed as previously described^20^. Whole cell lysates from cultured cells are were harvested in cell lysis buffer containing 20 mM Tris-HCl (pH 7.5), 150 mM NaCl, 1% Triton X-100, 1 mM EDTA, 1 mM EGTA, 2.5 mM sodium pyrophosphate, 1 Mm *β*-Glycerolphosphate, 50 mM NaF, 1 mM Na3VO4 and supplemented with protease inhibitor cocktail (#P8340, Sigma-Aldrich) for 30 min at 4 °C. Then the cell lysates were centrifuged at 4 °C (12, 000 rpm) for 15 min and protein extract supernatant was collected. Protein concentrations were determined with the Bradford protein assay kit (#500-0006, Bio-rad) using a Beckman DU-800 spectrophotometer (Fullerton, CA). Total cell lysates (20–30 μg) were separated by SDS-PAGE and transferred to nitrocellulose membrane (Bio-rad).The membranes were then blocked with diluted Odyssey® blocking buffer (#927-40000, LI-COR Biosciences, Lincoln, NE) for 1 h. The membranes were incubated with appropriate primary antibodies overnight at 4 °C and then washed with 1 X Tris Buffered Saline with 0.1% Tween-20 (TBST) for 3 times. The primary antibodies include human VCAM-1 (Santa Cruz, #sc-1504), human ICAM-1 (Santa Cruz, #sc-8439), mouse VCAM1 (R&D, #AF643) and GAPDH (EMD Millipore, #AB2302). Membranes were incubated with appropriate second antibodies at room temperature for 30 min. Odyssey Infrared Imaging System (LI-COR) was used to take images. Densitometric analysis of the blots was analyzed with NIH Image J software.

### Isolation of mouse lung endothelial cells

Mouse lung ECs were isolated as previously described^23^. *Klf2* ^+/−^ mice (B6; 129S4-*Klf2* ^*tm1.1Hhn*^/J, Stock# 026926) were obtained from The Jackson Laboratory. Briefly, lungs from three *Klf2* ^+/−^ or three *Klf2* ^+/+^ mice were minced into pieces. The lung pieces were digested using pre-warmed (37 °C) type I collagenase (#9001-12-1, Gibco) in a cell incubator at 37 °C for 45 min. The cell suspension was then filtered and centrifuged at 1200 rpm. The precipitates were resuspended in PBS containing BSA and penicillin/streptomycin and added CD31 (BD Pharmingen™, #553370) coated Dynabeads™ (Invitrogen) to incubate on a rotor at room temperature for 15 min. The cells in EP tubes were put on Magnetic Separation Rack and washed with growth medium (DMEM + 20%FBS + penicillin/streptomycin + heparin) for 4 times. Cells were resuspended in growth medium and grown in 0.1% gelatin coated 6-well plate. When the cells approached confluence, CD102 (BD Pharmingen™, #553326) coated Dynabeads™ sorting were performed. Then, the isolated cells were used to do the experiments. All animal procedures conformed to the Guideline for the Care and Use of Laboratory Animals published by the U.S. National Institutes of Health and were approved by the Institutional Animal Care and Use Committee of the University of Rochester Medical Center.

### *In vitro* anti-inflammatory assay

The anti-inflammatory effect assay was performed as follow. HCAECs or lung ECs were plated in 6-well plate and treated with vehicle (DMSO) or TA (10 µM) for 12 h. Recombinant human Tumor necrosis factor (TNFα) (R&D Systems, #210-TA) or murine TNFα (#315-01 A, PeproTech, USA) at a final concentration of 10 ng/ml was added for 3 h for qPCR assay or 6 h for WB assay, respectively. Then the cells were washes with PBS.

Total RNA were extracted as described in qPCR section. The mRNA expression levels of VCAM-1, ICAM-1, KLF2 and GAPDH were detected. The specific sequences of primers of quantitative real-time PCR were designed as follows: hKLF2: sense, 5′-CACGCACACAGGTGAGAA-3′, antisense, 5′-ACAGATGGCACTGGAATGG-3′; hVCAM-1: sense, 5′-TCAGATTGGAGACTCAGTCATGT-3′, antisense, 5′-ACTCCTCACCTTCCCGCTC-3′; hICAM-1, sense, 5′-GGCCGGCCAGCTTATACAC-3′, antisense, 5′-TAGACACTTGAGCTCGGGCA-3′; hET-1: sense, 5′-AAGGCAACAGACCGTGAAA-3′, antisense, 5′- GTCTTCAGCCCTGAGTTCTTT-3′; mKLF2: sense, 5′-CGTACACACACAGGTGAGAAG-3′, antisense, 5′-TGTGTGCTTTCGGTAGTGG-3′; mVCAM-1: sense, 5′-ACTCCCGTCATTGAGGATATTG-3′, antisense, 5′- TGACAGTCTCCCTTTCTTTGAG-3′; mGAPDH: sense, 5′-AACAGCAACTCCCACTCTTC-3′, antisense, 5′- CCTGTTGCTGTAGCCGTATT-3′.

Total proteins were extracted as described in Western blot part. The protein expression levels of human VCAM-1, human ICAM-1, mouse VCAM1 and GAPDH were detected by LI-COR.

### Cell adhesion assay

Monocyte adhesion to ECs was performed as previously described^15^. HCAECs seeded in 6-well plates were pretreated with vehicle or TA (10 µM) for 12 h. Recombinant human TNFα at a final concentration of 10 ng/ ml was added for 6 h. Then 0.5 ml (at a density of 5–7 × 10^6^/ml) THP-1 cells were added and incubated for 30 min. Then the cells were gentle washed with Meso Endo Cell Growth Media for 3 times to remove non-adherent THP-1 cells. The pictures were taken by Zeiss Axiovert 40 C microscope (magnification: 10 × ) with a Canon A640 digital camera (Canon USA Inc) with. The numbers of each image adherent cells were counted.

### siRNA knockdown assay

HUVECs were plated in 6-well plates. The cells were transfected with siControl (control siRNA, 20 nM, #AM4611, Thermo Fisher Scientific) or siKLF2 (KLF2 siRNA, 20 nM, #E006928, GE Dharmacon) in opti-MEM using RNA^MAX^ (Thermo Fisher Scientific). After 6 h, the medium were changed with cell culture medium with 10%FBS and incubated for 48 h. Then the cells were treated with TNFα or TA (10 µM) as described in cell adhesion assay.

### Statistical analysis

Data were analyzed by GraphPad Prism 5 software (GraphPad Software, Inc). Statistical comparisons and analyses between 2 groups were performed by 2-tailed, paired Student’s *t* test or one way ANOVA. Data were presented as mean ± SEM. *P* < 0.05 was considered statistically significant.

## Results

### Drug screening identifies TA as an activator of KLF2 expression

**Figure 1:**
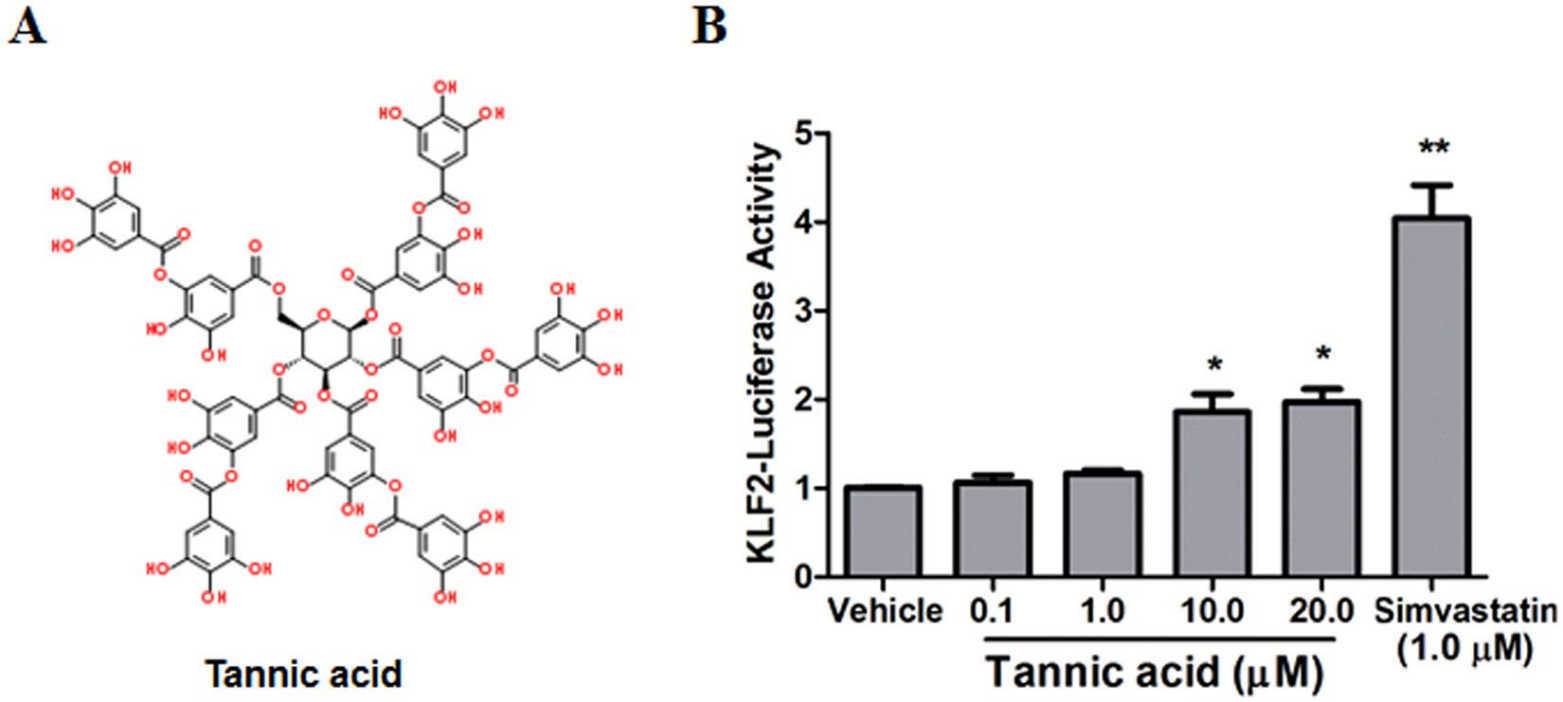
Identification of TA as a KLF2 activator. (**A**) Chemical structure of the TA. (**B**) KLF2 luciferase activity was analyzed in COS-7 cells transfected with KLF2-luciferase plasmids treatment with DMSO (vehicle), TA (0.1, 1.0, 10 and 20 μM) or simvastatin (1.0 μM). **P < 0.01, Student’s *t* test. Values represent mean ± SEM; n = 4.

**Figure 2:**
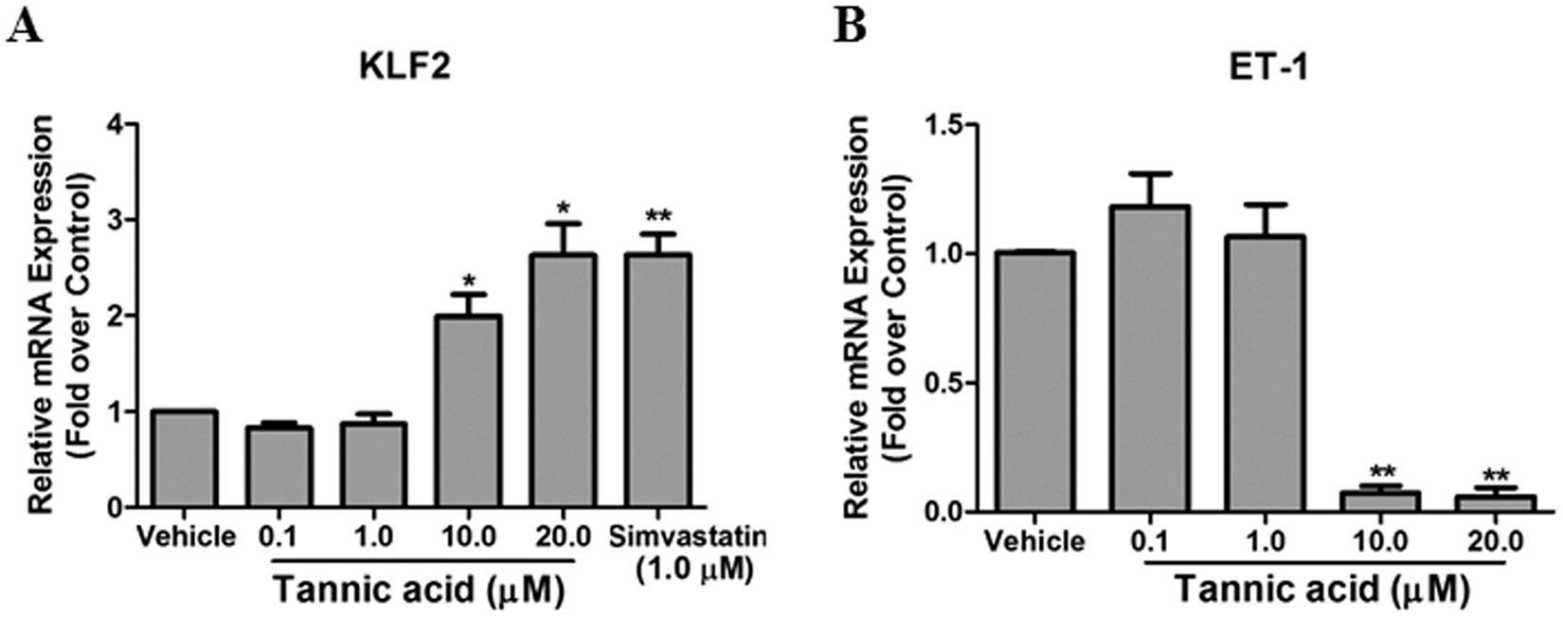
TA induced KLF2 expression in HCAECs. (**A**,**B**) HCAEC cells were treated with DMSO, TA (0.1, 1, 10 and 20 μM), or simvastatin (1.0 μM) for 24 h, then KLF2 (**A**) and ET-1 (**B**) mRNA expression was detected by qPCR. **P < 0.01, *P < 0.05 (TA or vs vehicle), Student’s *t* test. Values represent mean ± SEM; n = 4.

KLF2 promoter luciferase assays in COS-7 cells were performed as we described in methods section. Among the positive hit compounds, we observed that TA significantly increased KLF2 promoter luciferase activity at 5 µM (data not shown). The structure of TA is shown in Fig. 1A. Dose dependent assay showed that TA significantly increased KLF2 promoter luciferase activity at 10 and 20 µM (Fig. 1B). Simvastatin (1 µM), which was previously reported as a KLF2 activator^21, 24^, was used as a positive control. Next, the effect of TA on KLF2 was then confirmed by qPCR assay. KLF2 downstream target gene ET-1 was also examined. As shown in Fig. 2A, TA could increase KLF2 mRNA expression in HCAECs in a concentration-dependent manner. Meanwhile, TA significantly decreased ET-1 mRNA expression at 10 and 20 μM (Fig. 2B). Overall, our data suggest that TA is a KLF2 activator.

### TA induced KLF2 expression via the ERK5-MEF2 pathway

**Figure 3:**
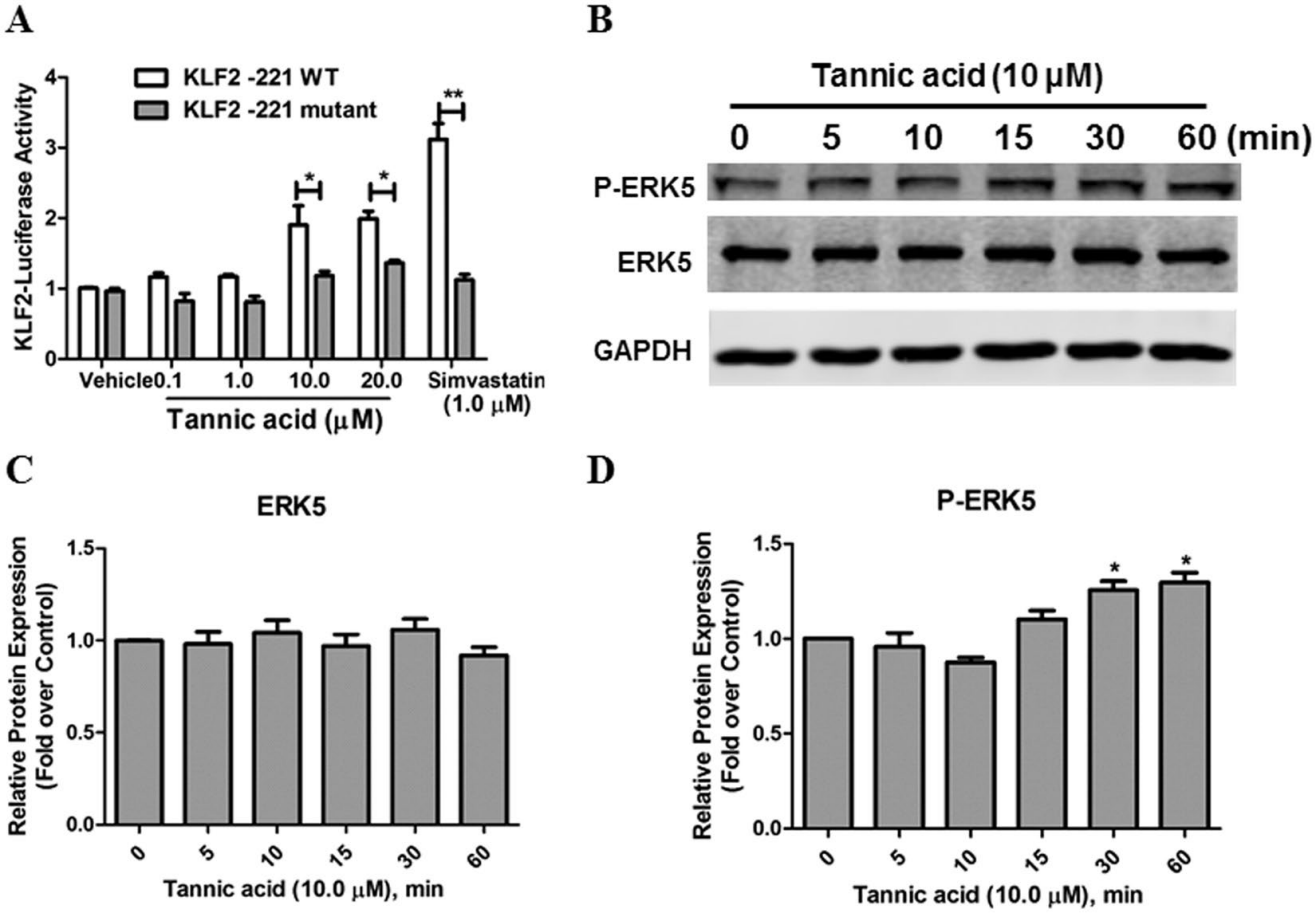
The ERK5/MEF2 pathway is involved in TA-induces KLF2 expression. (**A**) COS-7 cells were transfected with KLF2 -221 WT or KLF2 -221 mutant plasmids, treated with TA (10.0 µM), DMSO (vehicle) or simvastatin (1.0 µM, positive control) for 24 h, and luciferase activities were then detected. *P < 0.05. n = 3. (**B**–**D**) HCAECs were pretreated with TA (10.0 µM) for 0, 5, 10, 15, 30 and 60 min, respectively, then protein expression of phospho-ERK5, ERK5 and GAPDH were determined by Western blotting. *P < 0.05. n = 3.

To further investigate the signaling pathway leading to KLF2 upregulation by TA, the role of the two known upstream signaling molecules in KLF2 regulatory network, namely, ERK5 and MEF2 were assessed^7, 25^. Previous studies have shown that the transcription factor MEF2 is necessary for the induction of KLF2 by biomechanical and other types of stimuli^7, 21, 25^, therefore, we first investigated whether MEF2 was required for the TA-mediated increase in KLF2. Using the KLF2 -221 WT Luc promoter fragment which contains a single consensus MEF binding site, the KLF2 inductive effect of TA was totally maintained (Fig. 3A). To assess the importance of this site in TA-mediated induction of the KLF2 luciferase activity, we used the mutant in which MEF2 binding sites was disrupted in the context of the KLF2 -221 WT Luc construct. As shown in Fig. 3A, mutation of the MEF binding site almost completely abolished the TA-mediated induction of the KLF2 luciferase activity. In this experiment, simvastatin was used as a positive control, which could strongly induce KLF2 activity in KLF2 -221 WT Luc promoter plasmid but totally lost in KLF2 -221 mutant Luc promoter plasmid (Fig. 3A). These data implicate that TA induces the KLF2 promoter activity through MEF2 binding.

MEF2 transcription factor is one downstream target of ERK5, and ERK5 was required for laminar flow-induced expression of KLF2 in HUVECs^13^. To ask whether ERK5 are involved in TA-induced KLF2 expression, HCAECs were treated with TA and then the levels of ERK5 phosphorylation and total ERK5 were examined. As shown in Fig. 3B–D, TA induced ERK5-phosphorylation in a time-dependent manner without affecting total ERK5 expression. These results suggest that the ERK5-MEF2 dependent pathway is likely to mediate TA-induced upregulation of KLF2 in ECs.

### TA attenuated monocyte adhesion to endothelium and exhibited strong anti-inflammatory effect in ECs

**Figure 4:**
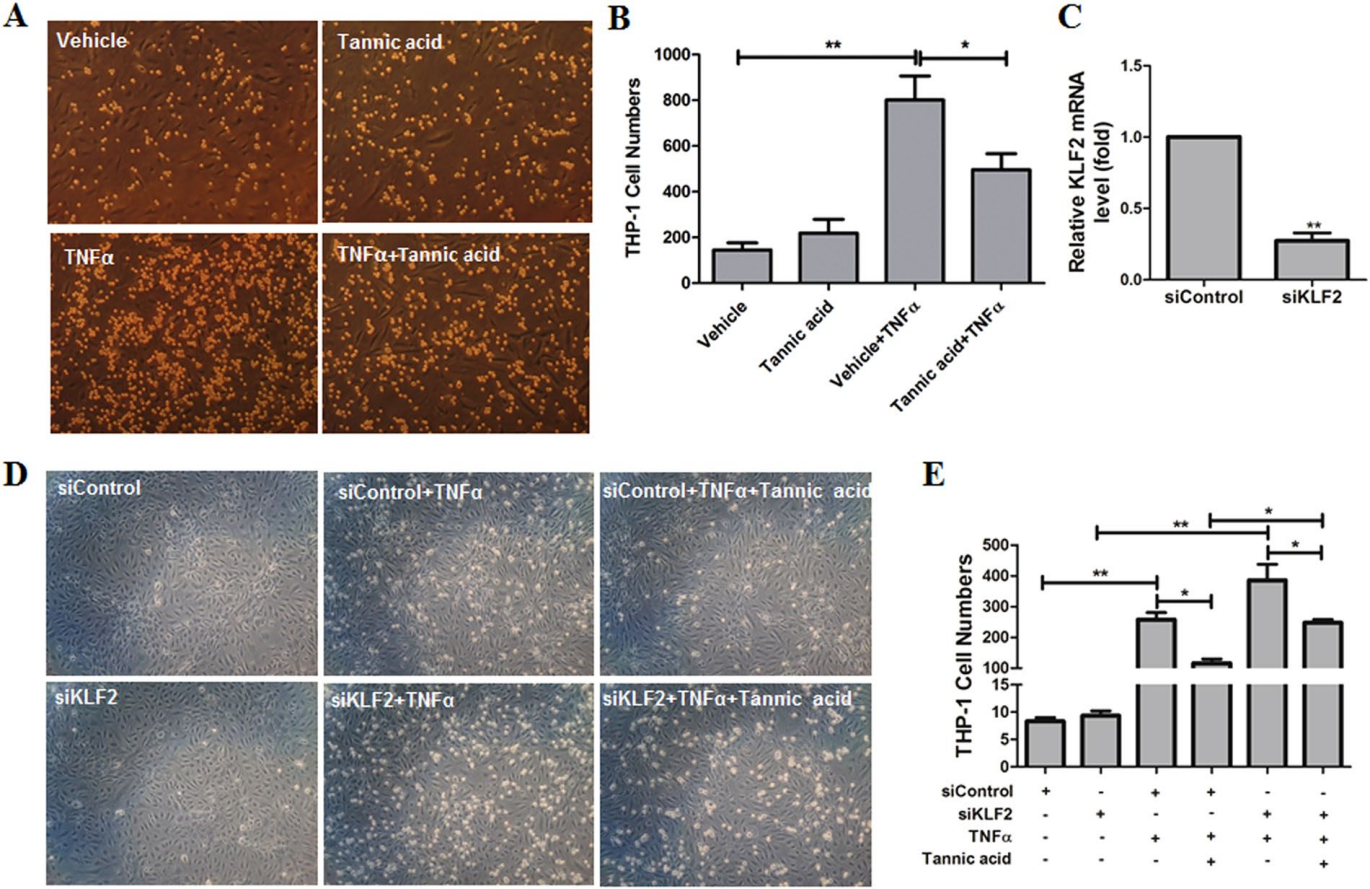
TA attenuated monocyte adhesion to ECs. (**A**,**B**) HCAECs were pretreated with vehicle (DMSO) or TA (10.0 µM) for 12 h, and then exposed to TNFα (10 ng/ml) or vehicle (PBS) for an additional 6 h. Then, THP-1 monocytes were added for 30 min. (**A**) Images were taken from representative optical fields showing endothelial cells (cobblestone shape) and adhering THP-1 monocytes (small, round cells) in the co-culture. (**B**) THP-1 cells in panel A were counted and statistically analyzed. *p < 0.05, **p < 0.01, n = 4. (**C**–**E**) HUVECs were transfected with siRNA (control siRNA) or siKLF2 (KLF2 siRNA) for 48 h. Then the cells were pretreated vehicle (DMSO) or TA (10.0 µM) for 12 h, and then exposed to TNFα (10 ng/ml) or vehicle (PBS) for an additional 6 h. Then, THP-1 monocytes were added for 30 min. (**C**) The levels of KLF2 mRNA in ECs treated with control siRNA and KLF2 siRNA were analyzed by q-PCR. (**D**) Images were taken from representative optical fields showing endothelial cells (cobblestone shape) and adhering THP-1 monocytes (small, round cells) in the co-culture. (**E**) THP-1 cells in panel C were counted and statistically analyzed. One way ANOVA was used to analyze the data. *p < 0.05, **p < 0.01, n = 3.

KLF2 is an anti-inflammatory molecule^26^, and has an important role in maintaining endothelial function. TNFα-induced monocyte adhesion to ECs was then performed to evaluate the anti-inflammatory effect of TA in HCAECs and HUVECs. As shown in Fig. 4A,B, TNFα significantly induced monocyte adhesion to HCAECs compared with vehicle, while TA treatment significantly reversed TNFα-induced monocyte adhesion (Fig. 4A,B). In order to exclude cell viability affects monocyte adhesion by TA. We performed cell viability assay in HCAECs and HUVECs to evaluate the possible toxicity of TA. We found that TA at 10 µM did not cause obvious toxicity at the same condition with cell adhesion assay (data not shown).

To ask whether the inhibitory effect of TA on monocyte adhesion is dependent on KLF2, we used KLF2 siRNA to knockdown KLF2 in ECs and examined its effect on monocyte adhesion. The efficiency of KLF2 siRNA (20 nM) to knockdown KLF2 in endothelial cells was analyzed by qPCR, and the results showed that about 70% KLF2 knockdown was achieved (Fig. 4C). When cells were transfected with control siRNA, TNFα significantly induced monocyte adhesion to HUVECs, and TA treatment significantly attenuated TNFα-induced monocyte adhesion (Fig. 4D,E), which was in consistent with Fig. 4A,B. When cells were transfected with KLF2 siRNA, the inhibitory effects of TA on TNFα-induced monocyte adhesion to endothelial cells was significantly reduced compared that with control siRNA treatment (Fig. 4D,E). Our results suggest that TA represses TNFα-induced monocyte adhesion at least in part via KLF2.

**Figure 5:**
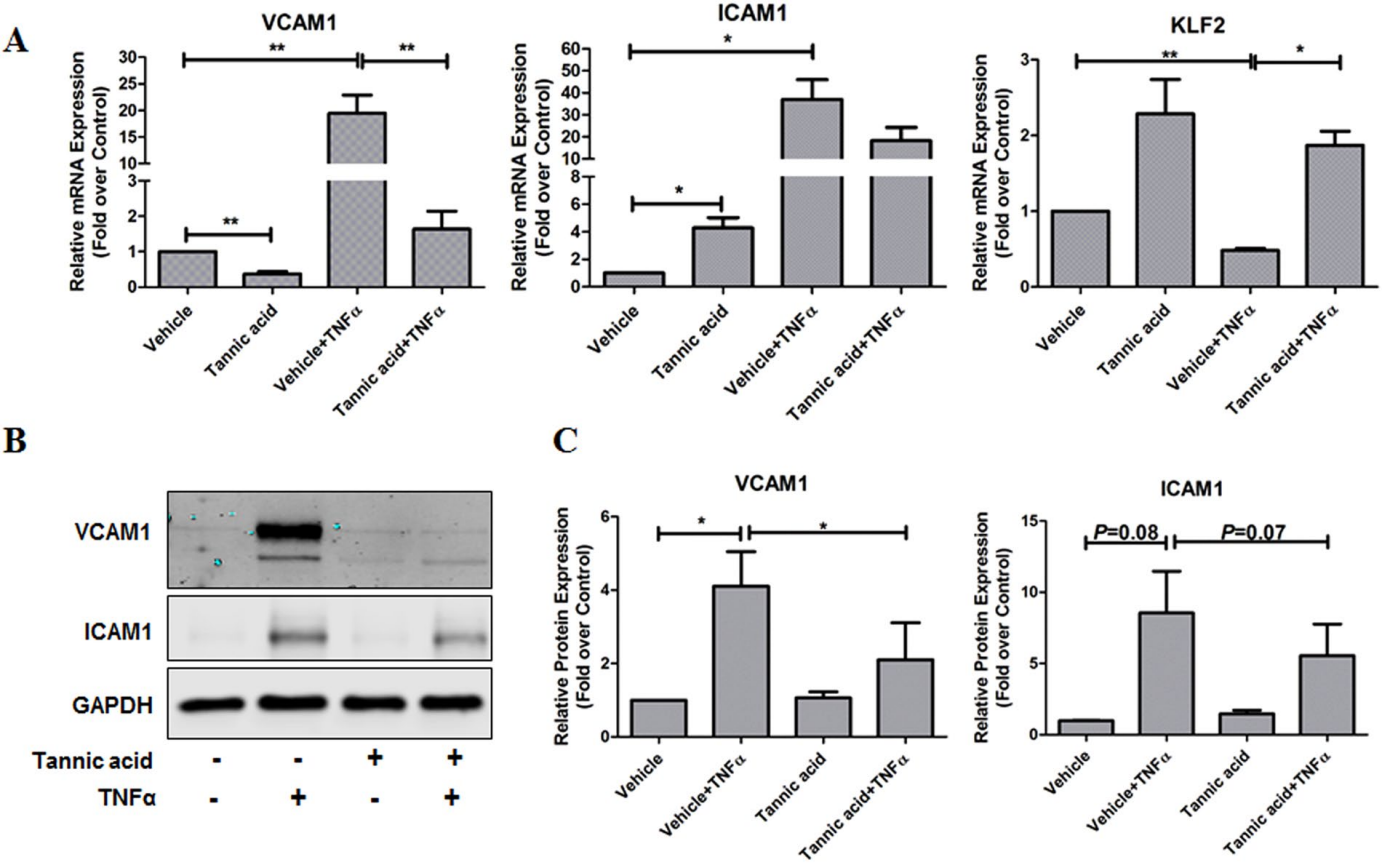
TA decreased TNFα-stimulated inflammatory response in HCAECs. (**A**) HCAECs were treated as described in Fig. 3 except for the exposure of TNFα for 3 h, then mRNA expression of VCAM1 and ICAM1 were determined by qPCR. Values are mean ± SEM, *p < 0.05, **p < 0.01, n = 5.B.HCAECs were treated as described in Fig. 4, then protein expression of VCAM1 and ICAM1 were determined by Western blot. (**C**) Quantification of panel B, values are mean ± SEM, *p < 0.05, **p < 0.01, n = 3.

To investigate the underlying molecular mechanisms by which TA attenuates monocyte adhesion to ECs, we examined the pro-inflammatory vascular cellular adhesion molecule-1 (VCAM1) and intercellular adhesion molecule-1 (ICAM1) mRNA and protein expression. As shown in Fig. 5A,B, TA significantly decreased TNFα-induced VCAM1 mRNA and protein expression, while the effect of TA on TNFα-induced ICAM1 mRNA and protein expression was not significant (5A-B). Meanwhile, TA protection against TNFα-induced monocyte adhesion was associated with a significant increase in expression of anti-inflammatory molecules KLF2 (Fig. 5C). Our results suggest that TA represses TNFα-induced monocyte adhesion via KLF2-dependent VCAM1 downregulation.

### The anti-inflammatory effect of TA is KLF2-dependent

**Figure 6:**
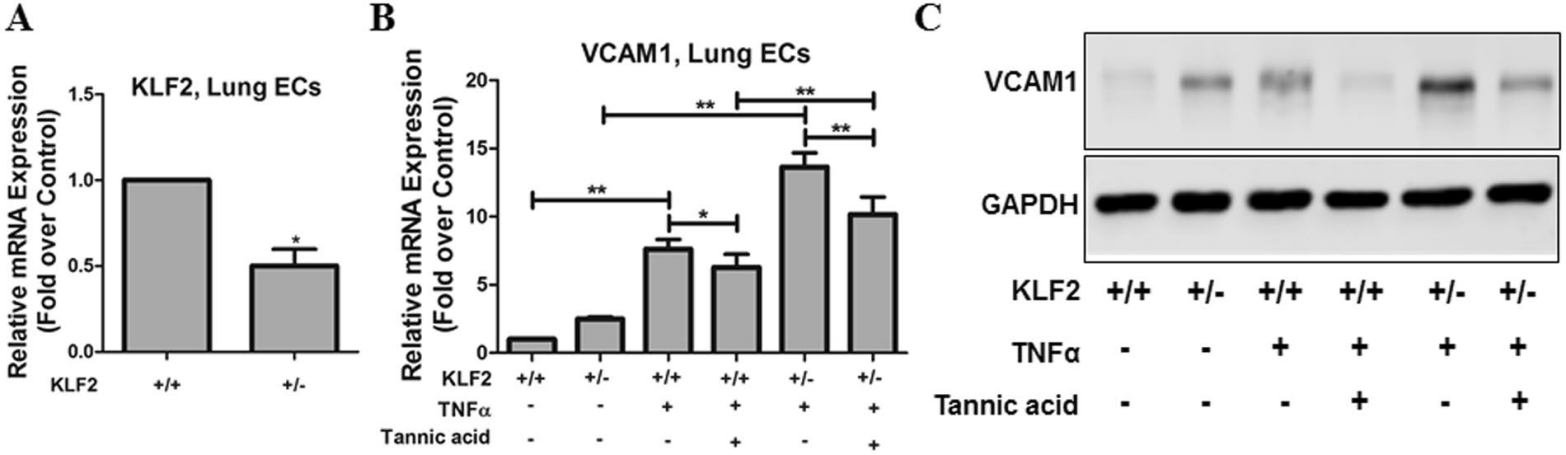
TA inhibited vascular inflammation via KLF2. Mouse lung ECs were isolated from *Klf2* ^+/+^ or *Klf2* ^+/−^ mice as described in methods. Pooled lung ECs from 3 mice with identical genotype were included in each group. (**A**) The levels of KLF2 mRNA expression in isolated lung endothelial cells from *Klf2* ^+/+^ and *Klf2* ^+/−^ mice were analyzed by qPCR. (**B**) Lung ECs were treated with or without TA for 12 h and stimulated with mouse TNFα for additional 3 h. qPCR was performed to detect VCAM1 mRNA expression. Statistical comparisons and analyses between 2 groups were performed by 2-tailed, paired Student’s *t* test. *P < 0.05, **P < 0.01, n = 5. (**C**) Lung ECs were treated with or without TA for 12 h and stimulated with mouse TNFα for additional 6 h. Western blot assays were performed to examine VCAM1 and GAPDH protein expression.

Next, we asked whether the anti-inflammatory effect of TA is KLF2-dependent. Because systemic KLF2 knockout was embryonically lethal^2^, *Klf2* ^*+/−*^ mice were used to determine KLF2-dependency of TA. Lung ECs from *Klf2* ^+/−^ and *Klf2* ^+/+^ mice were isolated and then stimulated with TNFα in the presence or absence of TA. As shown in Fig. 6, KLF2 expression in lung ECs from *Klf2* ^+/−^ mice was significantly decreased compared that from *Klf2* ^+/+^ mice (Fig. 6A). VCAM1 mRNA and protein expression was greatly induced by TNFα in *Klf2* ^+/−^ and *Klf2* ^+/+^ mouse lung ECs (Fig. 6B,C). TA significantly decreased VCAM1 mRNA and protein expression in KLF2^+/+^ mouse lung ECs. However, in lung ECs from *Klf2* ^+/−^ mice, the inhibitory effects of TA on TNFα-induced VCAM1 expression was partially reversed (Fig. 6B,C). These data demonstrate that the anti-inflammatory effect of TA was KLF2-dependent.

**Figure 7:**
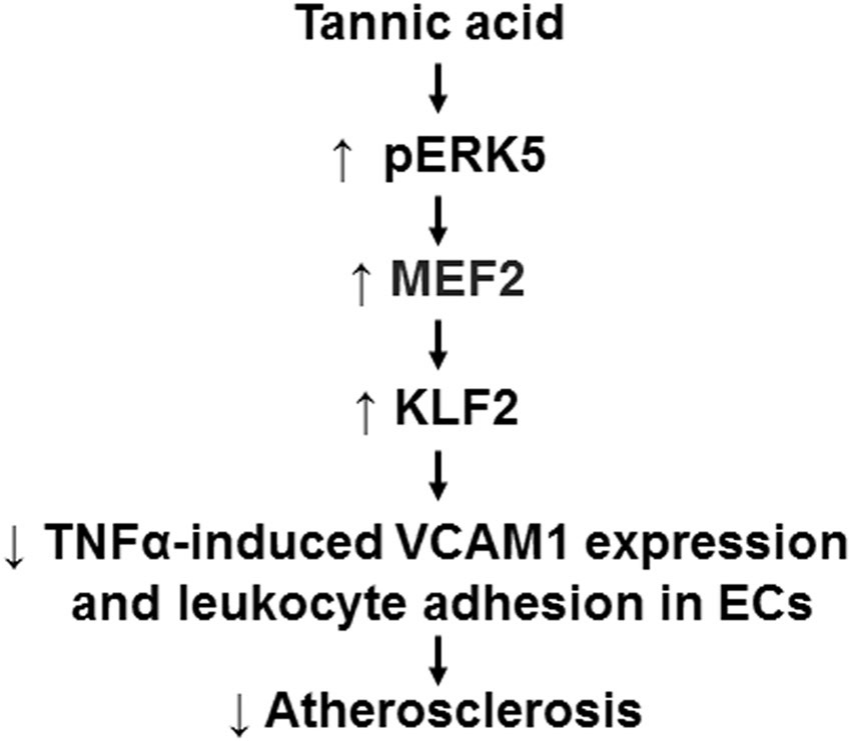
A working model depicting TA-mediated vasoprotective effects via a KLF2-dependent mechanism. Kruppel-like factor 2 (KLF2), tannic acid (TA), myocyte enhancing factor 2 (MEF2), extracellular-signal related kinase 5 (ERK5), phosphorylation (p-ERK5), vascular cell adhesion molecule 1 (VCAM1), endothelial cells (ECs), tumor necrosis factor alpha (TNFα).

Taken together, our data indicated that TA had potent anti-inflammatory effects in ECs. The schematic representation of the protective effect of TA on vascular endothelial inflammation is summarized as Fig. 7. TA induced KLF2 expression via the ERK5-MEF2 pathway, then decreased TNFα-induced monocyte adhesion through increased KLF2 expression and decreased VCAM1 expression, thus had an anti-inflammatory effect in ECs. Our study suggests to exploit TA as an effective plant-derived polyphenol that limits endothelial inflammation-associated cardiovascular diseases.

## Discussion

The central finding of this study is that TA induces KLF2 expression and attenuates TNFα-induced inflammation and monocyte cell adhesion in endothelial cells. To the best of our knowledge, our study is the first to demonstrate that KLF2 is critically involved in the effect of TA to prevent TNFα-induced endothelial inflammation.

Mounting evidence support that KLF2 is an anti-inflammatory and anti-atherosclerotic molecule. Thus, modulating KLF2 expression or activity could be a new therapeutic strategy for inflammatory-related disease such as atherosclerosis. Until now, there are some small molecules which have been reported to regulate KLF2 expression or activity. It has been reported that statins induce the KLF2 expression via MEF2^21^. Statins induce eNOS and thrombomodulin and exert endothelial atheroprotective effects dependent on transcriptional regulator KLF2, and thereby provides a novel mechanism for the beneficial effects of statins in cardiovascular disease^21, 24^. Moreover, it has been shown that the sirtuin 1 (SIRT1) activator resveratrol increases the expression of KLF2 via MEK5/MEF2 pathway in human endothelial cells, which helps us further understand the role of the SIRT1 activators in regulation of endothelial dysfunction-related cardiovascular disease and aging^25^. In addition, rapamycin, an mTOR inhibitor, could induce the expression and activity of KLF2, which might counteract coronary endothelial dysfunction^27, 28^. Taken together, these studies demonstrate that KLF2 may be a novel molecular target for modulating endothelial function. In this study, we performed high throughput screening and identified TA is a new KLF2 activator. We further showed that TA inhibited endothelial inflammation through upregulation of endothelial KLF2 expression and hence downregulation of adhesion molecule VCAM1 expression. Our results provide new insight into the mechanisms whereby TA attenuates vascualr inflammation and atherosclerosis.

TA is a plant-derived polyphenol that has been of great interest for many years. Polyphenolic compounds as important plant-derived dietary components have antioxidant and/or free radical-scavenging properties *in vitro* and play an important role in the prevention of coronary artery disease^29^. Among many studies, extracts from plants were most used. Choi group showed that 0.02% dietary TA has health-promoting effects by alleviating hepatic lipogenesis and atherogenesis in *ApoE* ^−/−^ mice through increasing liver peroxisome proliferator-activated receptor α (PPARα) expression^19^. In our study, we show that the vasoprotective effects of TA is mediated through activating KLF2, which provide mechanistic insights into vascular benefits of TA. Multiple factors and signaling pathways are involved in the regulation of KLF2 including shear stress, statins, phosphoinositide-3-kinase (PI3K)-dependent/Akt independent pathway and AMPK-dependent MEK5/ERK5/MEF2 signaling pathway^7, 30, 31^. Jain group has demonstrated that statins induced KLF2 luciferase activity via MEF2 binding site^21^. In this study, using KLF2 -221 WT and KLF2 -221 mutant plasmids, we found that simvastatin indeed induced KLF2 expression totally dependent on MEF2 binding site. Compared with simvastatin, TA also significantly increased KLF2 expression through MEF2. In addition, we observed that TA increased ERK5 phosphorylation. Therefore, it is likely that TA induced KLF2 expression in part through the ERK5/MEF2 pathway. Further studies are needed to prove the involvement of MEK5/ERK5/MEF2 in TA-induced KLF2 expression and anti-inflammatory effect. In addition, it warrants further investigation to see whether there are some other mechanisms including AMPK activation act synergistically in the process of TA-induced KLF2 expression.

KLF2 can differentially regulate endothelial genes, thus, it was considered as a key “molecular switch” governing endothelial function in vascular health and disease^9, 10, 21^. The observations presented here reveal that TA is a novel KLF2 activator and suggest that KLF2 could serve as a promising therapeutic target for the treatment of endothelial inflammation-associated cardiovascular disease.
